# Thyroid abscess: a rare complication following fine needle aspiration

**DOI:** 10.1093/jscr/rjaf829

**Published:** 2025-10-17

**Authors:** Caitlin Sorour, Andrew S Beatty, Roderick Borrowdale

**Affiliations:** Department of General Surgery, Redcliffe Hospital, Anzac Avenue, Redcliffe 4020, Queensland, Australia; Department of General Surgery, Cairns Hospital, The Esplanade, Cairns, 4870, Queensland, Australia; Department of General Surgery, Redcliffe Hospital, Anzac Avenue, Redcliffe 4020, Queensland, Australia; Northside Clinical School, School of Medicine, The University of Queensland, St Lucia 4072, Queensland, Australia; Department of General Surgery, Redcliffe Hospital, Anzac Avenue, Redcliffe 4020, Queensland, Australia

**Keywords:** thyroid, abscess, FNA

## Abstract

Abscess is a rare pathology to affect the thyroid gland owing to several protective anatomical and physiological factors. Anatomical abnormalities of the thyroid, underlying thyroid pathology and an immunocompromised state have been known to be the leading risk factors associated with thyroid abscesses with only a few case reports noted secondary to trauma and iatrogenic causes. This is a rare case of a thyroid abscess in an immunocompetent adult presenting following a fine needle aspiration (FNA) performed for investigation of a thyroid nodule. She was treated conservatively with antibiotics and ultrasound guided aspiration and recovered without further reoccurrence. This case outlines a rare complication of FNA which is a commonly performed investigation used in the workup for thyroid disease and highlights the importance of a timely diagnosis and appropriate management.

## Introduction

Thyroid abscesses are rare, representing ˂0.7% of surgically managed thyroid pathologies [1]. This is largely due to the anatomical properties of the thyroid which include the fibrous capsule and fascial planes that separate the thyroid from its surrounding structures. Additionally, the thyroid’s iodine-rich content which is bactericidal in nature with extensive lymphatic drainage make it very difficult for infections to seed to the thyroid [2, 3].

A thyroid abscess typically presents with pain and swelling of the anterior neck with associated fevers. Diagnosis is determined with a combination of clinical examination, blood work including inflammatory markers, imaging (most commonly ultrasonography) and aspiration for microscopy and culture [2, 4]. The accurate and timely diagnosis of thyroid abscesses is important due to the significant complications including thyroid storm, sepsis, internal jugular vein thrombosis and airway compression [5, 6].

Due to its rarity, literature regarding thyroid abscesses is limited to case studies and case series with even less focus placed on iatrogenic causes of thyroid abscess [2, 7]. We present a case of an immunocompetent 39-year-old lady who developed a thyroid abscess 2 weeks following a FNA of her thyroid for investigation of a thyroid nodule.

## Case report

A 39-year-old female presented to the emergency department with a 2-week history of increasing pain and swelling to her left anterior neck. Prior to this she had undergone a FNA of her thyroid for investigation of a self-detected thyroid nodule; the cytology returned benign. She was complaining of subjective fevers and associated mild dysphagia as the swelling and pain had increased over the 2 weeks. She denied any odynophagia, dysphonia, drooling, or shortness of breath. Her past medical history included a previous left hemithyroidectomy for multinodular goitre 15 years prior. Otherwise, she had no significant medical history and was taking no regular medications.

On examination she was clinically stable, able to talk in full sentences without any stridor or evidence of airway compromise and her vitals were stable. She had a firm palpable neck mass of the left anterior neck measuring approximately 3 × 3 cm. There was no cervical lymphadenopathy or overlying erythema.

**Figure 1:**
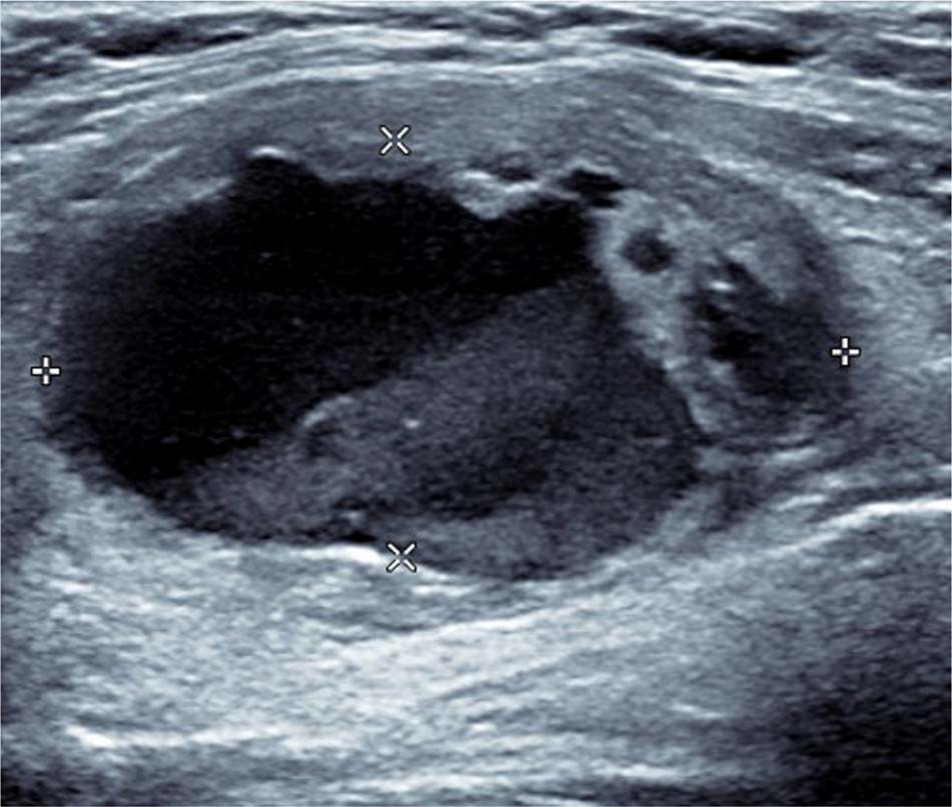
USS of the thyroid demonstrating the cystic lesion in the left thyroid gland.

**Figure 2:**
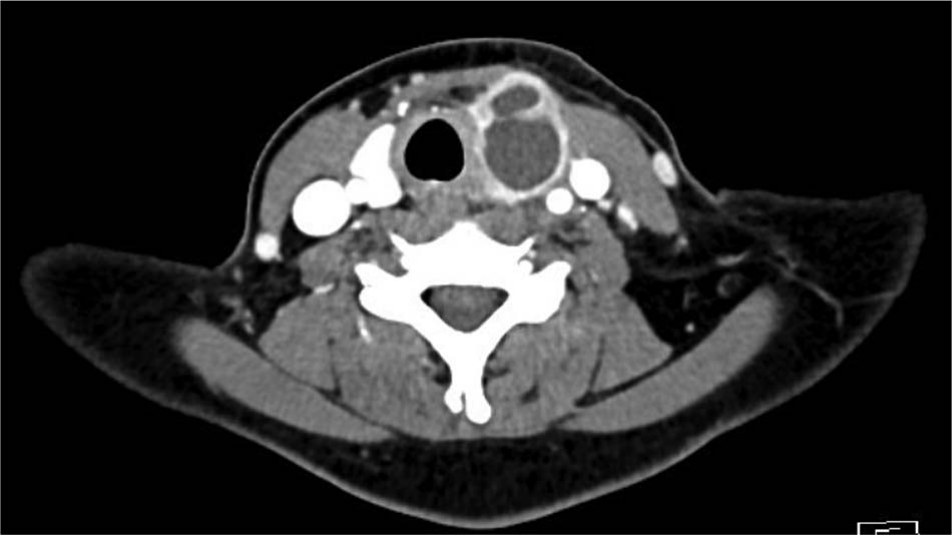
Axial portal venous phase CT of the neck demonstrating the cystic lesion in the left thyroid gland.

**Figure 3:**
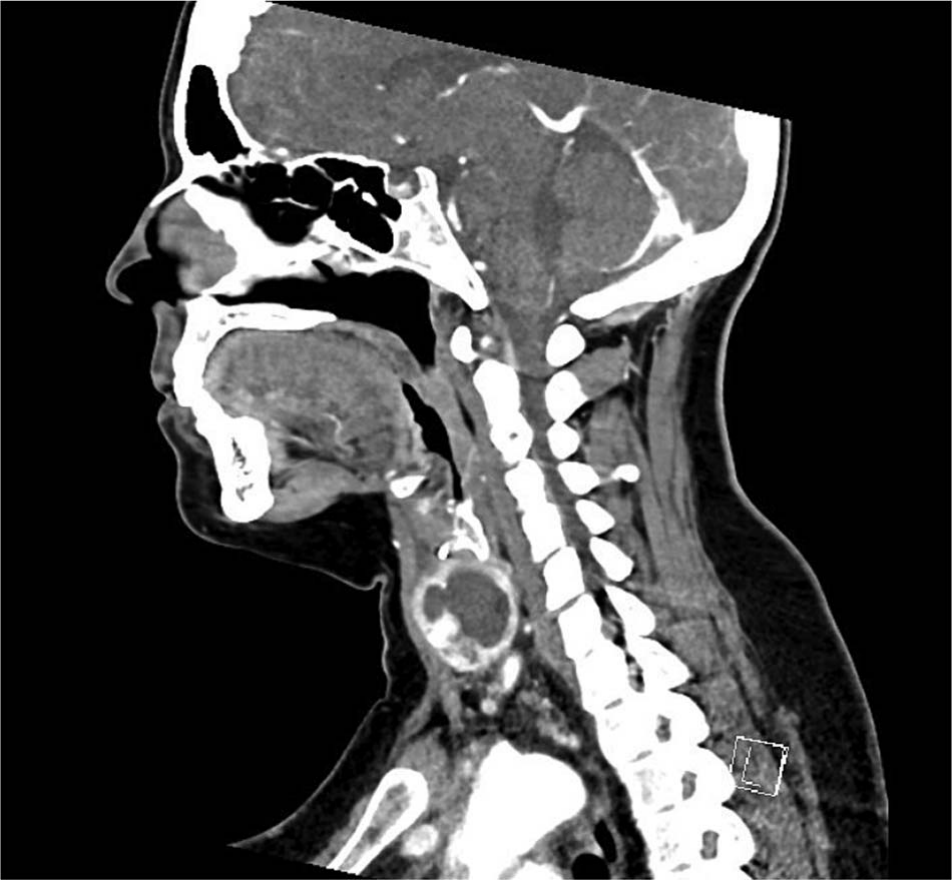
Sagittal portal venous phase CT of the neck demonstrating the cystic lesion in the left thyroid gland.

Bloods demonstrated elevated inflammatory markers with a neutrophil count of 7.53 and C-reactive protein of 137. Her biochemistry panel including thyroid function tests were within normal limits. An ultrasound (USS) was performed which showed a cystic/solid nodule in the left thyroid gland that measured 30 × 42 × 22 mm with an estimated volume of 14 ml (Fig. 1). A computed tomography (CT) scan was conducted to ensure no airway compression, it demonstrated the abscess to measure 32 × 26 × 32 mm (Figs 2 and 3). There was no tracheal compression but there was mild inflammatory stranding and hyperenhancement of the nodule consistent with infection.

She was admitted under the surgical team and commenced on broad spectrum intravenous antibiotics (piperacillin-tazobactam) and dexamethasone to reduce the risk of development of airway oedema or compromise. Within 24 h of presentation she proceeded for an ultrasound guided aspiration of the abscess which was aspirated to completion. The fluid on microscopy was found to have 2+ leucocytes but no organisms were grown, likely as the patient had already commenced antibiotic therapy prior. She remained for close observation for another 48 h following aspiration but improved without any clinical concerns. She was stepped down to oral cephalexin as discussed with the microbiology team and she completed a total course of 10 days. She was reviewed in the outpatient department and has fully recovered without re-accumulation of the abscess nor needing any further treatment.

## Discussion

Thyroid abscesses due to their scarcity are often an overlooked differential however, a timely diagnosis is important in preventing significant complications. The thyroid is an organ naturally resistant to infection due to its anatomical and physiological advantages and as such thyroid abscesses are a rare occurrence. The most common organisms responsible are staphylococcus and streptococcus species though gram negative have been documented; hence, until organisms have been cultured broader coverage is recommenced [7, 8].

The aetiology of thyroid abscesses varies with age with anatomical abnormalities including the presence of a pyriform sinus or a persistent thyroglossal duct being the most common in children. In adults, an immunocompromised state or underlying thyroid pathology such as goitre or malignancy are more common risk factors—as seen in this case study with the patient’s prior history of multinodular goitre [7, 9]. Trauma and iatrogenic causes of thyroid abscesses have been noted in the past with multiple cases of abscess formation following fine needle aspiration noted in the literature [7, 10–12]. Most of these cases, however, were noted to occur in immunocompromised individuals rather than our case of a well immunocompetent patient. Management typically involves a trial of conservative management with intravenous antibiotics and USS guided aspiration of the abscess. Refractory or higher severity cases often require surgical management with either incision and drainage of the abscess or a partial or total thyroidectomy hence these patients should be monitored in a hospital with the capabilities for surgical management if required [7, 10–12]. Regardless, iatrogenic causes of thyroid abscess remain a rare cause with find needle aspiration widely considered a safe diagnostic technique for thyroid nodules with almost no associated morbidity and mortality [10].

## Conclusion

This case highlights a rare complication associated from a thyroid aspiration. Patients should also be consented for this rare outcome and educated about signs and symptoms to ensure prompt assessment by medical staff to prevent the progression of any significant complications.
